# Weaning outcomes after early vs. late tracheostomy in severe burn injury: a retrospective single-center study

**DOI:** 10.1186/s12893-026-03535-6

**Published:** 2026-01-24

**Authors:** Julien-Moritz Thielmann, Wolfram Heitzmann, Michael Ried, Jan Akkan, Paul Christian Fuchs, Jennifer Schiefer, Till Markowiak

**Affiliations:** 1 Department of Plastic, Reconstructive, Hand and Burn Surgery, Cologne-Merheim Medical Center (CMMC), Witten/Herdecke University (Campus Cologne-Merheim), Ostmerheimer Straße 200, Köln, 51109 Germany; 2 Department of Thoracic Surgery, University Hospital Regensburg, Regensburg, 93053 Germany

**Keywords:** Tracheotomy, Weaning, Mechanical Ventilation, Burns, ICU, Airway-Management

## Abstract

**Background:**

Patients with severe burn injuries often require prolonged mechanical ventilation (MV). The benefit of an early tracheostomy (ET; ≤ 10 days after injury) to reduce MV duration and facilitate weaning remains unclear. This study aimed to compare weaning outcomes between patients undergoing ET and late tracheostomy (LT; > 10 days after injury) in a high-volume burn center.

**Materials and methods:**

We conducted a retrospective analysis of 67 patients admitted to our burn intensive care unit from January 2015 to June 2024. The patients were assigned to two groups based on the timing of the tracheostomy: ET (*n* = 52) and LT (*n* = 15). Endpoints of the study were the influence of tracheostomy timing on the time to milestones in the weaning process (CPAP, intermittent breathing without MV, decannulation) and ICU length of stay.

**Results:**

The mean age was 52.9 (± 19.7) years. The ABSI-scores were similarly distributed, with medians of 9 in the ET group and 8 in the LT group. Inhalation trauma was present in 55.2% of patients across both groups (ET *n* = 29 (55.8%); LT *n* = 8 (53.3%)). Regarding weaning milestones, no significant differences were observed in the median time to first intermittent breathing without MV (ET 15 days vs. LT 20 days; *p* = 0.47) or median time to decannulation (ET 33 days vs. LT 69 days; *p* = 0.28). However, patients in the ET group reached CPAP significantly earlier than those in the LT group (ET 7 days vs. LT 13 days; *p* < 0.001). Overall, in-hospital mortality rate was 43.3% (ET 46.2% vs. LT 33.3%; *p* = 0.38).

**Conclusion:**

This study found no substantial benefit associated with early-onset tracheostomy in patients with severe burn injuries. While ET resulted in significantly earlier progression to CPAP-mode, it did not expedite overall weaning success or reduce time to decannulation. While the sample size may limit sensitivity to detect smaller effects, the findings provide insight into weaning dynamics following early versus LT in burn patients. These findings underscore the need for standardized, prospective evaluation in this population.

## Background

Severe burn injuries often require prolonged mechanical ventilation (MV) due to airway compromise, inhalation injury, and systemic inflammatory responses. Worldwide, around 11 million burn injuries require medical care annually, with over 180.000 deaths predominantly occurring in low- and middle-income countries [1]. Among these patients, the need for advanced airway management, including tracheostomy, is one of the most critical and discussed components of care [2]. Thus, tracheostomy is a common intervention in patients with severe burn injuries, particularly those with inhalation trauma or extensive burns involving the head and neck, when prolonged ventilation support is expected [3]. The procedure facilitates airway clearance, reduces the risk of ventilator-associated pneumonia (VAP), and enables early weaning from MV in critically ill patients [4].

Despite its prevalence, the timing of tracheostomy in burn patients vary significantly [5]. This variability reflects differences in healthcare systems, resource availability, and clinician preferences [6]. For instance, in high-income countries, where access to intensive care units (ICUs) is robust, tracheostomy rates and timing decisions are often guided by evidence-based protocols. In contrast, low-resource settings may delay tracheostomy due to a lack of surgical expertise or equipment, potentially impacting patient outcomes [6]. However, even in countries where sufficient medical resources are available and both early and LT are generally feasible, the timing of such procedures remains a topic of ongoing debate [2]. ET is proposed to expedite weaning from MV, reduce sedation requirements, and potentially improve patient outcomes [7]. Conversely, LT, performed after the initial stabilization phase, allows clinicians to better assess the need for prolonged airway support while avoiding unnecessary procedures in patients with a good prognosis for spontaneous recovery [8].

Advocates of ET emphasize its potential benefits, citing evidence from non-burn critically ill cohorts where ET has been linked to reduced ICU length of stay and a lower incidence of VAP [5]. On the other hand, ET is not without risks. Surgical complications such as bleeding, infection, and tracheal stenosis can be significant, particularly in the acute phase of burn care when patients often have coagulation abnormalities or systemic inflammation [9–11]. Additionally, the placement of a tracheostomy in the early phase of care might interfere with burn wound management in the neck region [12]. In contrast, LT may avoid unnecessary procedures in patients who recover sufficiently to be weaned from MV without a tracheostomy [13]. However, prolonged intubation carries its own risks, including increased incidence of laryngeal injury and discomfort [13]. Delayed tracheostomy may also be associated with prolonged ICU stays and higher rates of complications related to prolonged MV, such as VAP [14].

The decision to perform tracheostomy in burn victims is often influenced by preclinical factors, including the extent of burn injury, presence of inhalation trauma, and initial stabilization efforts. Burn patients frequently present with multisystem trauma, requiring clinicians to prioritize interventions that stabilize hemodynamics and address other life-threatening injuries before considering tracheostomy [15]. Additionally, factors such as the severity of airway compromise and patient comorbidities may guide the timing of the procedure [15]. Despite the critical importance of the timing of tracheostomy, evidence specific to burn patients is limited. Most studies on tracheostomy timing are conducted in mixed ICU populations, with findings extrapolated to burn patients. Retrospective analyses of burn patients have yielded conflicting results regarding the benefits of ET versus LT, underscoring the need for further research in this field to inform clinical practice [2, 16, 17].

In this context, our study investigates the impact of tracheostomy timing on weaning outcomes in patients with severe burn injuries. By analyzing data from a high-volume burn center, we aim to clarify whether ET facilitates earlier liberation from MV compared to LT and whether this has implications for overall patient outcomes. These findings will contribute to the body of evidence guiding the timing of tracheostomy in this unique patient population.

## Patients and methods

### Study population

This retrospective, single-center study included patients admitted to our burn-ICU due to a burn trauma of at least 15% total body surface area (TBSA) between January 2015 and June 2024 who underwent either surgical tracheostomy or percutaneous dilatational tracheostomy (PDT). Patients were stratified into two groups according to the timing of tracheostomy: the ET group (*n* = 52), comprising patients who underwent tracheostomy ≤ 10 days post-injury, and the LT group (*n* = 15), which included those who received tracheostomy > 10 days following the burn event. This study received approval from our local institutional ethics committee (registration number: S-27/2025) and was conducted in alignment with the principles outlined in the Declaration of Helsinki.

### Timing, decision-making and method of tracheostomy

**Fig. 1:**
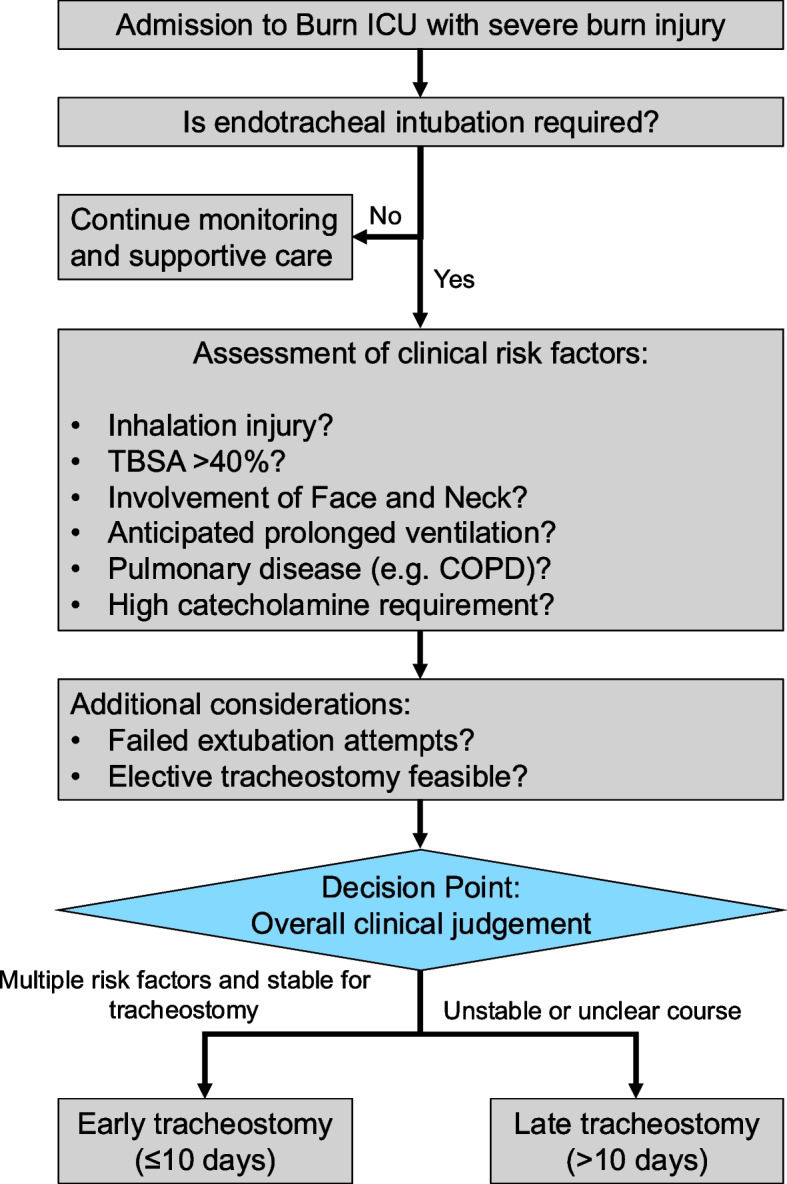
Flowchart of clinical decision-making for ET versus LT in severe burn injury

The timing and method of tracheostomy were not guided by a fixed institutional protocol but was instead determined by individualized clinical judgment. Each case was jointly assessed by the attending burn surgeon and intensivist, considering factors such as hemodynamic stability, airway status, and the anticipated duration of mechanical ventilation. In general, tracheostomy was performed electively once patients were stable enough to tolerate the procedure and if prolonged ventilation was expected. As detailed in Fig. 1, tracheostomy timing was determined through a structured multidisciplinary evaluation—guided by predefined clinical criteria and applied on a case-by-case basis in a high-acuity burn ICU. Additionally, we extracted predefined clinical covariates from the notes to contextualize timing decisions: vasopressor use within 24 h, renal replacement therapy, sepsis, wound infection, failed extubation, anterior neck involvement, and time from trauma to first necrosectomy.

### Data acquisition

Data extraction was performed through review of electronic medical records, operative reports, and inpatient documentation. The collected data included demographic variables such as age, sex, and body mass index (BMI), as well as burn severity indicators, including TBSA affected and Abbreviated Burn Severity Index (ABSI). The presence of inhalation injury and pre-existing pulmonary conditions, such as bronchial asthma or chronic obstructive pulmonary disease (COPD), was also recorded. Further variables encompassed for example the type of tracheostomy performed (open surgical vs. PDT) and the number of surgical interventions. Outcome parameters included the incidence of VAP, time to first spontaneous breathing without mechanical ventilation, duration to tracheostomy decannulation or closure, time to first conversion to continuous positive airway pressure (CPAP), overall length of hospital stay, and in-hospital mortality. VAP was diagnosed in accordance with IDSA/ATS-Guidelines (Infectious Diseases Society of America/American Thoracic Society) (2016) and institutional protocols, based on the presence of new or progressive radiographic infiltrates in combination with at least two of the following: fever > 38.3 °C, leukocytosis or leukopenia, purulent respiratory secretions, and microbiological confirmation from endotracheal aspirate or bronchoalveolar lavage.

### Study endpoints

The primary endpoint of this study was to evaluate the weaning process from MV in relation to the timing of tracheostomy in patients with severe burn injuries. To provide a comprehensive assessment, weaning was evaluated using predefined milestones, including time to first conversion to CPAP-mode, time to first spontaneous breathing without MV and time to decannulation. Furthermore, the ICU length of stay was evaluated. Time to CPAP initiation was measured as the interval from trauma to the start of CPAP support. Time to first spontaneous breathing without MV was defined as the duration from trauma to the first successful spontaneous breathing trial without mechanical ventilatory support ≥ 30 min, reflecting the initial transition from full ventilatory support to partial respiratory autonomy. Time to decannulation was recorded as the time from tracheostomy placement to the successful removal of the tracheostomy tube, representing the restoration of sufficient airway patency and respiratory function to maintain adequate ventilation without artificial airway support.

### Tracheostomy technique

Tracheostomy represents a pivotal intervention in the management of patients with severe burn injuries, particularly those necessitating prolonged mechanical ventilation or airway protection. Two predominant techniques are utilized: surgical tracheostomy and PDT.

Open surgical tracheostomy involves a 2–3 cm transverse incision between the cricoid cartilage and suprasternal notch. The dissection must preserve critical structures, including the brachiocephalic trunk, recurrent laryngeal nerve, and thyroid gland [16, 18]. In open tracheostomy, division of the thyroid isthmus can be necessary, as it may be required to adequately expose the tracheal rings, particularly in anatomically complex patients. A tracheal incision, typically at the second or third ring, allows for direct cannulation and a secure airway [18]. Compared to PDT, this method provides a wider stoma, facilitating tube exchanges and long-term airway management. To promote epithelialization, a tracheal mucosal flap may be anastomosed to the skin. For long-term use, a stable epithelialized tracheostoma can be created by suturing a flap of tracheal mucosa to the skin, which reduces the risk of wound complications [17].

PDT is a minimally invasive procedure routinely employed in intensive care settings, valued for its flexibility and reduced risk of wound infections. The procedure is typically conducted between the second and fourth tracheal rings. Under local anesthesia and guided by bronchoscopy, a percutaneous puncture of the tracheal lumen is performed, followed by the introduction of a guidewire to facilitate progressive dilation. A tracheostomy tube is then placed, with its correct positioning confirmed via end-tidal CO₂ monitoring and bronchoscopy [19]. PDT is anatomically not suitable for all patients, particularly those with anatomical abnormalities, obesity or edema that may complicate access to the trachea. On the downside a recognized limitation of PDT is the potential difficulty of re-cannulation following accidental early decannulation and may close if the tube is removed for an extended period [17, 19].

### Statistical analysis

Data collection and statistical analyses were conducted using IBM SPSS Statistics, Version 29 (IBM Corporation, Armonk, NY, USA). Categorical data were described as absolute numbers and percentages, and group comparisons were performed using the chi-squared test of independence or Fisher's exact test. Interval-scaled data were reported as mean ± SD or median (IQR), depending on the data distribution, and compared using either Student's t-test or the Mann–Whitney U-test. The Shapiro–Wilk test was employed to assess the normality of the data distribution. The Kaplan–Meier method was utilized to analyze weaning progression, defined as the time from the date of trauma to the achievement of each weaning milestone. Patients who died before reaching a defined milestone were censored at the time of death in the Kaplan–Meier analysis, and the corresponding values were marked as missing in the dataset. The log-rank test was applied to evaluate the impact of tracheostomy timing on weaning progression. Additionally, a multivariate Cox regression model was developed to identify independent risk factors for prolonged weaning. Correlations between ICU length of stay and period to weaning milestones were assessed using Spearman’s rank correlation coefficient. A *p*-value < 0.05 was considered statistically significant for all analyses.

## Results

### Demographics

The mean age of patients in the ET cohort (49.3 years ± 18.6) was significantly lower than that of the LT group (65.7 years ± 18.1; *p* = 0.003). No significant variations were observed in the gender distribution between the two groups (*p* = 0.33). BMI (*p* = 0.88) and affected TBSA (*p* = 0.29) values were comparable between the groups. Additionally, the ABSI-scores were similarly distributed across both groups (ET: median 9 (IQR 3.75), LT: median 8 (IQR 3), *p* = 0.98). Inhalation injury was observed in 55.5% of the entire cohort. Specifically, 29 patients (55.8%) in the ET group and 8 patients (53.3%) in the LT group sustained inhalation trauma. Pulmonary comorbidities, including asthma (ET: *n* = 2 (3.8%), LT: *n* = 1 (6.7%)) and COPD (ET: *n* = 4 (7.7%), LT: *n* = 1 (6.7%)), were present at similar rates across both groups (*p* = 0.22 and *p* = 0.89). The patient characteristics are shown in Table 1.

**Table 1:** Demographics

		All patients	ET (≤ 10 days)		LT (> 10 days)		***p***-value	
		(*n* = 67)	(*n* = 52)		(*n* = 15)			
Age [years], mean (± SD)		52.9 (± 19.7)	49.3 (± 18.8)		65.7 (± 18.1)		0.003*	
Male gender, n (%)		47 (70.1)	38 (73.1)		9 (60)		0.33	
BMI [kg/m^2^], median (IQR)		26.4 (7.1)	26.4 (7.9)		26.7 (6.9)		0.88	
TBSA [%], median (IQR)		35 (32)	36.5 (33.6)		33.5 (15.5)		0.29	
ABSI-Score, median (IQR)		8 (3)	9 (3.75)		8 (3)		0.98	
Initial PaO₂/FiO₂, median (IQR)		380 (230)	374.6 (212.2)		462.9 (271.5)		0.58	
Inhalation trauma, n (%)		37 (55.2)	29 (55.8)		8 (53.3)		0.86	
Burns involving the neck, n (%)		44 (65.7)	35 (67.3)		9 (60)		0.59	
Pre-existing pulmonary conditions, n (%)		10 (14.9)	8 (15.4)		2 (13.3)		0.84	
· Asthma, n (%)		3 (4.5)	2 (3.8)		1 (6.7)		0.22	
· COPD, n (%)		5 (7.5)	4 (7.7)		1 (6.7)		0.89	

*ABSI* Abbreviated Burn Severity Index, *BMI* Body mass index, *COPD* Chronic obstructive pulmonary disease, *ET* Early-onset tracheostomy, *FiO₂* Fraction of inspired oxygen, *IQR* Interquartile range, *LT* Late-onset tracheostomy, *PaO₂* Oxygen partial pressure, *SD* Standard deviation, *TBSA* Total body surface area burned

^*^Statistically significant

### Clinical data

**Fig. 2:**
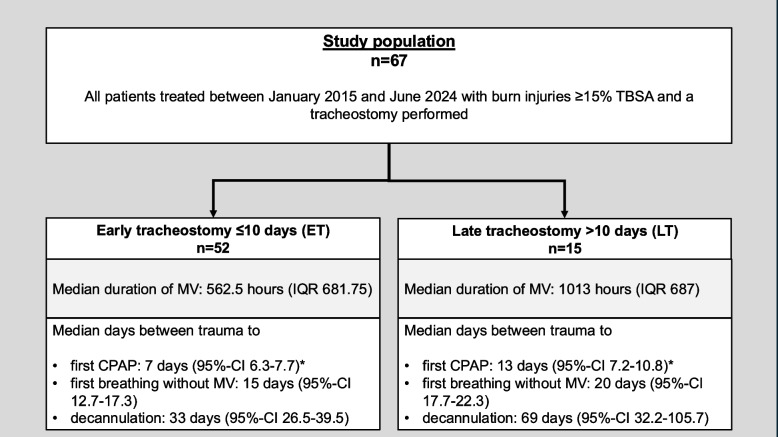
Flowchart of the study population outlining ET and LT cohorts with key weaning milestones. *Statistically significant

Table 2 and Fig. 2 present the clinical data of the cohort. The tracheostomy approach was predominantly surgical in both groups (ET: *n* = 50; 96.2% versus LT: *n* = 13; 86.7%). Tracheostomy closure via surgical intervention was performed in 13 patients (25%) in the ET group, while no patients (0%) in the LT group underwent this procedure (*p* = 0.031). The period between trauma and first surgical necrosectomy was significantly longer in the LT group (3 days, IQR 2) compared to the ET group (2 days, IQR 3) with a *p*-value of 0.020.

**Table 2:** Clinical data

	All patients	ET (≤ 10 days)	LT (> 10 days)	***p***-value
	(*n* = 67)	(*n* = 52)	(*n* = 15)	
Failed extubation	3 (4.5)	3 (5.8)	0 (0)	1.0
Tracheostomy, n (%)	67 (100)	52 (100)	15 (100)	0.17
· PDT, n (%)	4 (6)	2 (3.8)	2 (13.3)	
· Surgical tracheostomy, n (%)	63 (94)	50 (96.2)	13 (86.7)	
Period between trauma and first necrosectomy [days], median (IQR)	2 (2)	2 (3)	3 (2)	0.020*
Catecholamine requirement in the first 24 h, n (%)	66 (98.5)	51 (98.1)	15 (100)	0.59
Surgical procedures, median (IQR)	6 (6)	6 (5.75)	6 (5)	0.63
VAP, n (%)	59 (88.1)	45 (86.5)	14 (93.3)	0.73
Wound infections, n (%)	57 (85.1)	46 (88.5)	11 (73.3)	0.15
Sepsis, n (%)	45 (67.2)	32 (61.5)	13 (86.7)	0.12
Dialysis, n (%)	11 (16.4)	11 (21.2)	0 (0)	0.06
Surgical tracheostomy closure, n (%)	13 (19.4)	13 (25)	0 (0)	0.031*
Dysphagia, n (%)	32 (47.8)	23 (44.2)	9 (60)	0.38
Duration of MV [hours], median (IQR)	655 (669)	562.5 (681.75)	1013 (687)	0.07
ICU duration [days], median (IQR)	42 (41)	41 (38)	65 (55)	0.16
In-hospital mortality, n (%)	29 (43.3)	24 (46.2)	5 (33.3)	0.38

*ICU* Intensive care unit, *IQR* Interquartile range, *MV* Mechanical ventilation, *PDT* Percutaneous dilatational tracheostomy, *ET* Early-onset tracheostomy, *LT* Late-onset tracheostomy, *VAP* Ventilator-Associated Pneumonia

^*^Statistically significant

Furthermore, no significant differences in the total number of surgical procedures were observed, indicating comparable surgical burdens between the groups (*p* = 0.63). Throughout the study cohort, patients underwent six surgical interventions in the median, which included tangential necrosectomies, the application of various wound dressings (such as Dressilk © and Suprathel ©), skin grafting procedures utilizing multiple techniques—including Meek technique (micrografts) and mesh grafts—and the tracheostomies.

The incidence of VAP was similar between both cohorts (overall: *n* = 59 (88.1%), ET: *n* = 45 (86.5%), LT: *n* = 14 (93.3%)) (*p* = 0.73). Overall, in-hospital mortality rate was 43.3% (*n* = 29). There was no statistical difference between the ET group (46.2%; *n* = 24) and the LT group (33.3%; *n* = 5) (*p* = 0.38).

The duration of ICU length of stay did not show statistically significant differences between the ET and LT cohorts, with the ET group demonstrating a median stay of 41 days (IQR 38 days) compared to a median stay of 65 days (IQR 55 days) in the LT group (*p* = 0.16) (Table 2).

### Weaning

**Fig. 3:**
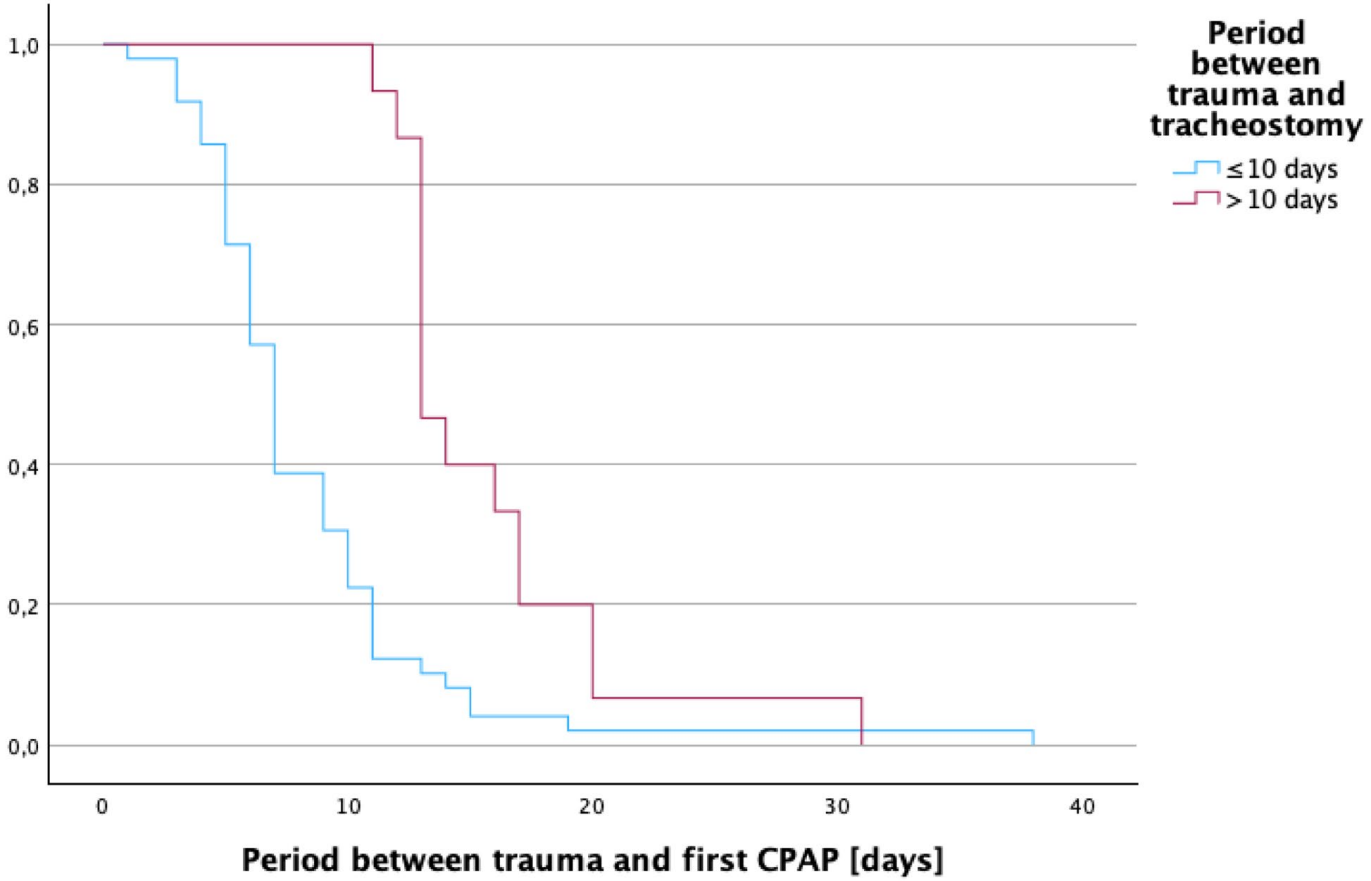
Kaplan–Meier analysis depicting time from injury to initial CPAP transition, comparing ET versus LT cohorts to evaluate the impact of tracheostomy timing on weaning dynamics. (*p* = 0.001)

In terms of weaning, Patients receiving tracheostomy within 10 days demonstrated a significantly faster progression to CPAP ventilation, with a median of 7 days (95% CI: 6.3–7.7), compared to 13 days (95% CI: 7.2–10.8) in the LT group (*p* < 0.001) (Fig. 3). However, no statistically significant differences were observed in the median time to routine humidified oxygen in low-flow oxygen therapy (ET: median 15 days (95%-CI: 12.7–17.3) versus LT: median 20 days (95%-CI 17.7–22.3); *p* = 0.47). Similarly, the median time to decannulation did not differ significantly between groups: 33 days (95% CI: 26.5–39.5) for the ET group versus 69 days (95% CI: 32.3–105.7) for the LT group (*p* = 0.28). The total duration of MV across the entire cohort was a median of 655 h (IQR: 669). Patients with ET had a median ventilation duration of 562.5 h (IQR: 681.75), while those with LT had a median of 1013 h (IQR: 687) (*p* = 0.07) (Table 2). Time from burn to CPAP, first routine humidified oxygen in low-flow oxygen therapy, and decannulation were all positively correlated with ICU length of stay (Spearman’s rho = 0.31, *p* = 0.013; rho = 0.52, *p* < 0.001; rho = 0.83, *p* < 0.001).

### Independent risk factors

A multivariable Cox proportional hazards regression model was performed to identify independent risk factors for prolonged weaning among patients with severe burn injuries. Specifically, age, gender, ABSI score, and preexisting pulmonary conditions were systematically analyzed in relation to the predefined key weaning milestones (Table 3). In addition, comparative analyses between the ET and LT cohorts were performed. The analysis revealed a significant association between a higher ABSI-score and an extended time to the initiation of first spontaneous breathing without mechanical ventilation (HR = 0.86, 95% CI: 0.75–0.98, *p* = 0.033), as well as a delay in decannulation (HR = 0.77, 95% CI: 0.63–0.94, *p* = 0.012). Additionally, age, gender and preexisting pulmonary conditions did not emerge as an independent predictor for prolonged weaning time.

**Table 3:** Cox regression analysis of independent risk factors

	Trauma to first CPAP-transition		Trauma to first breathing without MV		Trauma to decannulation	
	HR (95% CI)	*p*-value	HR (95% CI)	*p*-value	HR (95% CI)	*p*-value
Age	1.01 (0.99–1.02)	0.35	1.01 (0.99–1.03)	0.28	1.03 (0.99–1.05)	0.09
Gender	1.25 (0.7–2.3)	0.46	1.35 (0.65–2.82)	0.42	1.2 (0.49–3.03)	0.68
ABSI-Score	1.03 (0.93–1.1)	0.59	0.86 (0.75–0.98)	0.033*	0.77 (0.63–0.94)	0.012*
Pulmonary Conditions	1.39 (0.69–2.81)	0.35	1.66 (0.65–4.22)	0.29	0.79 (0.28–2.21)	0.65

*ABSI-Score* Abbreviated Burn Severity Index, *CPAP* Continuous Positive Airway Pressure, *HR* Hazard Ratio, *MV* Mechanical Ventilation, *95% CI* 95% Confidence Interval

^*^ Statistically significant

## Discussion

This study found no substantial benefit associated with ET in patients with severe burn injuries. Extensive research has explored the optimal timing and potential benefits of tracheostomy in critically ill patients requiring mechanical ventilation. Systematic reviews and meta-analyses of randomized controlled trials have consistently shown that ET is associated with reduced durations of mechanical ventilation and shorter ICU stays compared to LT. However, it is crucial to note that these studies primarily focused on general critical care populations and did not include patients with severe burn injuries [20–22]. Given the unique pathophysiological challenges associated with major burns—such as inhalation injury, airway edema, and complex wound management—findings from these general ICU populations cannot be directly applied to severely burned patients. The timing of tracheostomy in burn patients is a complex decision, influenced by a range of clinical factors, including the severity of burn injury and inhalation trauma, the presence of comorbidities, and the anticipated extent for surgical interventions.

In our study, we examined a cohort of 67 burn patients who underwent tracheostomy, a notably large sample size in comparison to existing studies in this field. For instance, Smailes et al. conducted a study involving 41 patients, investigating the role of ET in conjunction with active exercise programs for severe burn patients in the ICU [23]. Similarly, Mourelo et al. focused on a smaller cohort of 20 patients, specifically addressing the management of tracheostomy in thermal injury cases [9]. In contrast, Saffle et al. analyzed 44 burn patients to evaluate the impact of ET on outcomes [9].

The demographic and clinical data of the ET and LT groups were largely comparable. Although the LT group exhibited a significantly higher mean age compared to the ET group, no significant differences regarding gender distribution, BMI, or TBSA were observed between the groups. In particular, the ABSI-score, which reflect the severity of burn injury and predict overall prognosis, was evenly distributed. We analyzed clinical parameters potentially influencing the timing of tracheostomy in our retrospective approach, including early vasopressor use, inhalation injury, renal replacement therapy and sepsis. While no significant differences were observed between groups for these variables, the time from trauma to first necrosectomy was significantly longer in patients with LT. This observation is compatible with delayed tracheostomy occurring in patients who experienced a more protracted early clinical course — in our cohort reflected by a significantly longer interval from trauma to first necrosectomy in the LT group — where airway procedures were generally deferred until hemodynamic stabilization. These data are observational and do not establish causality, but they provide clinically relevant context for interpreting timing decisions. While several systemic severity indicators—such as ABSI, inhalation injury, and total number of surgical procedures—were included in our analysis, granular physiological data (e.g., SOFA scores) were not available for the entire cohort in a standardized format and thus could not be incorporated into multivariate models. This represents a limitation of the retrospective study design and should be addressed in future prospective research.

As no standardized protocol for tracheostomy timing was in place, the decision for early versus late tracheostomy reflected the dynamic assessment of each patient’s condition rather than adherence to predefined criteria. Hemodynamic instability, the need for multiple early surgeries, or severe systemic illness often delayed the procedure until stabilization was achieved. This individualized approach represents real-world burn ICU practice and, while limiting direct comparability with prospective standardized studies, provides valuable insight into how complex clinical judgment influences timing in this specific patient population.

Our findings indicate that while ET may lead to significant earlier CPAP progression, it does not substantially enhance overall weaning success or reduce the time to tracheostomy closure. While it is hypothesized that an earlier transition to CPAP ventilation could reduce the risk of VAP by enhancing pulmonary function and facilitating secretion management, this assumption remains largely unproven in general and is even less substantiated in the context of burn patients. The theoretical benefit of earlier CPAP initiation is based on its potential to minimize the duration of invasive mechanical ventilation, which is a known risk factor for pneumonia [24]. However, our data indicate that the incidence of VAP did not differ significantly between the ET and LT groups. Thus, while CPAP transition may improve respiratory management, its effect on VAP prevention remains uncertain and warrants further investigation. Furthermore, in patients with severe burn injuries, multiple factors, including wound healing, pain management, and infection control, significantly influence the weaning [25]. These elements may overshadow any isolated weaning benefit of ET, suggesting that tracheostomy timing alone is unlikely to be the primary determinant of weaning success in the population.

ET did not result in a significant reduction of the overall duration of MV. Similarly, the time from burn injury to the initiation of routine humidified oxygen in low-flow therapy remained comparable between groups, indicating that ET did not markedly accelerate the transition to fully spontaneous breathing. Moreover, the duration of artificial airway dependence, as reflected by the time to decannulation, was similar across cohorts. These findings stand in contrast to previous studies that have reported potential advantages of ET, particularly in critically ill patients, including those with severe burns. For example, Smailes et al. demonstrated that ET in burn patients was associated with a reduction in mechanical ventilation duration, and earlier initiation of active exercise, as well as improved functional outcomes at discharge [23]. The potential for earlier initiation of physiotherapy and speech therapy in ET patients is another purported advantage, particularly in cases involving inhalation trauma [9]. Similarly, the meta-analysis of Shan et al. reported significant reductions in both mechanical ventilation duration for patients undergoing ET [26]. Despite these reported benefits in the literature, our study did not observe comparable improvements. In our cohort, in-hospital mortality did not differ significantly between groups (ET 46.2% vs. LT 33.3%; *p* = 0.38). Given the limited sample size, the study is likely underpowered to detect differences in mortality.

The definition of ET varies across the literature, with cut-offs ranging from ≤ 7 to ≤ 14 days after intubation [23]. In our study, a 10-day cut-off was chosen, reflecting the definition of other significant studies in this field [27]. This approach aligns the definition of ET with the early stabilization phase while acknowledging variability in the literature [15].

While several studies, including the meta-analysis by Shan et al., have demonstrated a shorter ICU length of stay in ET cohorts, our analysis did not show a statistically significant difference between groups [26]. Nevertheless, previous research has suggested potential clinical advantages of ET—such as improved secretion management and reduced sedation requirements—which may still be relevant when interpreting our findings in the broader clinical context [23]. ICU length of stay is inherently multifactorial, influenced by burn severity, comorbid conditions, and individual recovery trajectories. Our analyses indicate that shorter ICU stays were associated with faster progression through key weaning milestones. Importantly, however, the data do not support the conclusion that the timing of tracheostomy alone was the decisive factor.

Furthermore, our findings emphasize the pivotal role of burn severity in the weaning process of mechanically ventilated burn patients. The association between high ABSI scores and delayed weaning suggests that systemic responses to severe burns may outweigh the influence of tracheostomy timing.

Inhalation trauma, which occurred in over half of the patients in our study, is a critical factor influencing respiratory morbidity and has been proven to contribute to prolonged mechanical ventilation and delayed weaning [28]. Inhalation injury occurs in approximately 10% to 20% of burn patients in general [29]. Notably, inhalation trauma is associated with higher rates of ET, as early airway intervention is often prioritized to prevent progressive airway edema and respiratory compromise. However, the evidence regarding the impact of ET on long-term outcomes in these patients remains unclear [28].

In our cohort, all patients received mechanical ventilation despite the absence of documented inhalation injury. This reflects the complex and multifactorial nature of respiratory support in severe burn trauma. Beyond inhalation-related airway compromise, several clinical factors may necessitate ventilatory support, including the extent and depth of burn injury, systemic inflammatory response, fluid shifts leading to pulmonary edema, sedation requirements, and airway protection during altered consciousness and intensive pain management. Notably, in the international LAMiNAR cohort of 160 burn patients, 74% were ventilated with lung-protective settings regardless of inhalation injury, and tidal volumes did not differ between those with and without documented inhalation trauma [30]. These findings underscore that respiratory support in this population often extends far beyond direct pulmonary injury and must be understood within the broader context of critical illness physiology.

The evidence supporting ET in non-burn populations should be interpreted with caution when considering its application to this distinct patient cohort. In line with this, a more cautious approach may be considered, favoring a strategy that prioritizes initial stabilization of the burn shock and the patient's overall hemodynamic status before proceeding with tracheostomy. This approach could also minimize potential unnecessary interventions and reduce the risk of complications associated with premature tracheostomies. This reflects a more conservative approach that seeks to balance burn recovery and respiratory care while avoiding premature tracheostomies. This is consistent with the growing body of evidence suggesting that tracheostomy timing in severely burned patients should be considered on an individual, case-by-case basis [15, 31].

While our study focused on weaning-related outcomes, it is important to acknowledge that earlier transition to CPAP after tracheostomy may carry additional benefits that are not routinely measured. A shorter duration of translaryngeal intubation can significantly affect patient comfort, facilitate earlier communication, and reduce the need for sedation—factors that are particularly relevant in the burn population. Moreover, prolonged translaryngeal intubation is associated with long-term complications such as dysphagia, impaired phonation, and laryngotracheal stenosis [32]. These sequelae are often underappreciated in the acute setting but may persist for years and severely impact quality of life. Awareness of these issues increased notably during the COVID-19 pandemic, when prolonged intubation was often favored over tracheostomy due to concerns about aerosol generation [33]. However, the clinical relevance of minimizing translaryngeal intubation extends well beyond that context. As emphasized by Brenner et al., a broader patient centered perspective that incorporates functional outcomes is essential to fully evaluate the implications of tracheostomy timing [32]. Postoperative dysphagia was systematically assessed in our cohort, revealing no significant differences between early and LT groups, while other complications such as hoarseness could not be reliably evaluated retrospectively.

While this study offers meaningful insights into the impact of ET in severe burn patients, certain limitations should be considered. The retrospective, single-center design may affect the generalizability of the findings, as patient management protocols and resources may differ significantly across burn centers. The small sample size results in wide confidence intervals within the Kaplan–Meier analyses, thereby reducing the precision and warrants cautious interpretation. Additionally, some physiologic parameters, including standardized oxygenation indices were not uniformly available, which constrained comprehensive adjustment for illness severity. Variability in clinical management practices, such as sedation strategies, physiotherapy and timing of surgical interventions, reflects real-world complexity but could not be fully standardized. Also, the absence of systematic follow-up regarding secondary outcomes—such as tracheal stenosis or vocal cord dysfunction and mortality—limits conclusions on potential long-term benefits or harms of ET and should be addressed in future longitudinal studies. Furthermore, the non-randomized group assignment may introduce selection bias. These considerations highlight opportunities for future prospective, multicenter studies to further refine understanding and optimize timing strategies for tracheostomy in this unique cohort. Finally, because tracheostomy timing was based on individualized clinical decisions rather than a predefined algorithm, variability in clinical reasoning may persist. Although we attempted to capture relevant clinical variables such as hemodynamic support, renal replacement therapy, infection, and time to first surgery, additional unmeasured factors may have influenced timing. Future prospective studies incorporating standardized documentation of the clinical rationale for tracheostomy timing will be essential to further clarify these decision processes.

## Conclusion

In conclusion, this study highlights the complexity of the decision-making process regarding tracheostomy timing in patients with severe burn injuries. Our findings suggest that ET does not offer a substantial advantage in terms of respiratory weaning or duration of mechanical ventilation. The first days after severe burn trauma are often characterized by hemodynamic instability, multiple surgical interventions of the skin and soft tissues, so that a tracheostomy before stabilization of the patient is a burden that, according to our data, should be critically evaluated. While the findings are suggestive of potential differences, conclusions regarding clinical relevance and ICU stay reduction should be interpreted with caution, as the limited sample size may preclude detection of more nuanced effects. These results highlight the urgent need for prospective, multicenter data to establish evidence-based recommendations on tracheostomy timing in burn patients; in the meantime, individualized multidisciplinary decision-making remains necessary in complex cases.

## Data Availability

The datasets generated are available from the corresponding author upon reasonable request.

## Abbreviations

ABSI: Abbreviated Burn Severity Index
BMI: Body Mass Index
CI: Confidence Interval
COPD: Chronic Obstructive Pulmonary Disease
CPAP: Continuous Positive Airway Pressure
ET: Early-Onset Tracheostomy
HR: Hazard Ratio
ICU: Intensive Care Unit
LT: Late-Onset Tracheostomy
MV: Mechanical Ventilation
PDT: Percutaneous Dilatational Tracheostomy
SD: Standard Deviation
TBSA: Total Body Surface Area
VAP: Ventilator-Associated Pneumonia
IQR: Interquartile Range
IDSA/ATS: Infectious Diseases Society of America / American Thoracic Society
